# A case report of cervical pregnancy after *in vitro* fertilization complicated by tuberculosis and a literature review

**DOI:** 10.1515/med-2020-0236

**Published:** 2020-11-09

**Authors:** Kun Liu, Xuehong Zhang, Xiaoling Ma, Xueling Jia, Xiaodong Zhao, Xia Yang, Yufeng Zhang

**Affiliations:** Reproductive Medicine Centre, The First Hospital of Lanzhou University, Lanzhou, 730030, Gansu, China

**Keywords:** infertility, tuberculosis, pregnant, treatment, IVF

## Abstract

Although female genital tuberculosis may lead to infertility, pregnancy is still possible, especially through *in vitro* fertilization (IVF). In this eventuality, even latent tuberculosis (TB) infection is prone to reactivate. Because some of the symptoms of TB overlap with those of pregnancy, diagnosis and treatment may be delayed. We report the case of a 30-year-old infertile woman with repeated genital tuberculosis (GTB) who underwent two laparoscopic surgeries and anti-TB treatments. The woman conceived through IVF and, unfortunately, a cervical pregnancy was diagnosed, together with a third recurrence of GTB. When the condition became stable after anti-TB treatment, the pregnancy was terminated using oral mifepristone in combination with an ultrasound-guided local injection of methotrexate. The gestational sac was expelled 4 days later with minimal blood loss. In view of the reciprocal influence and interconnection between IVF, pregnancy, and TB, we conducted a literature review to provide valuable information for early diagnosis and treatment, as well as for routine screening before IVF of TB in infertile patients.

## 1 Introduction

Most female genital tuberculosis (FGTB) cases are secondary to pulmonary tuberculosis (TB). Mycobacterium is commonly transmitted to plentiful vascular reproductive organs through blood routes, among which the fallopian tubes, endometrial, and ovarian are the most frequently infected sites. These sites appear not through only organic damage, but also through immune dysfunction, thus leading to infertility [1,2]. It was reported that FGTB accounts for 7–19% of infertility [3].

Due to diverse clinical manifestations and the lack of specific symptoms and signs, FGTB can be easily misdiagnosed. On the other hand, once the immune defense system is activated, infected *Mycobacterium tuberculosis* would enter a dormancy state and be retained for a long time. Conditions such as malnutrition, diabetes, or pregnancy can reduce the immune response, leading to the reactivation of latent infections and the development of active pulmonary TB and/or FGTB [4,5].

This paper reports a 30-year-old infertile woman with repeated genital tuberculosis (GTB) who underwent two laparoscopic surgeries and anti-TB treatments. The woman conceived through *in vitro* fertilization (IVF), and unfortunately, a cervical pregnancy was diagnosed together with a third recurrence of GTB.

Following the description of this case, we review the mutual relationship and influence of IVF, pregnancy, and TB, as well as cervical pregnancy diagnosis and treatment, aiming to provide clinicians with useful information to aid treatment decisions for such patients.

## 2 Case report

A 30-year-old female came to our practice complaining of primary infertility over the last 4 years. Her history indicated that in 2014, tuberculotic peritonitis had been diagnosed and treated laparoscopically, following which she received TB chemotherapy for 6 months. In 2016, the right Fallopian tube was laparoscopically resected and a pathology examination confirmed the tuberculotic nature of the lesions. Afterward, TB chemotherapy was reinstated for another year. Wishing to become pregnant, she requested IVF. Before carrying out the procedure, both a tuberculin skin test (TST) and chest computed tomography (CT) were carried out. The TST showed a water bubble after 72 h, and the CT scan showed streaking on the medial segment of the middle lobe of the right lung and a protuberance under an enlarged lymph node.

In view of her having twice received anti-TB treatment, it was decided not to repeat that treatment; ovarian stimulation was initiated following administration of a short-acting depot gonadotropin-releasing hormone analogue for pituitary suppression. Because of the husband's asthenospermia, the intracytoplasmic sperm injection technique was used. On the third day after fertilization, two grade II embryos [6] were transferred, and three others were frozen.

Eleven days after the embryo transfer, the patient complained of diarrhea. At that time, her serum hormone levels were as follows: β-human chorionic gonadotropin (β-HCG), 49.5 mlU/mL; oestradiol (*E*_2_) 80.8 pg/mL; and progesterone (*P*) 12.8 ng/mL The symptoms improved following intravenous fluids and antibiotics. On day 14 after the transfer, the patient presented with intermittent fever, from 37.8 to 38.5°C, and a cough; she again received symptomatic treatment. On day 18 the patient’s body temperature rose once more to 38.9°C. At this point serum, blood levels were β-HCG 371.6 mIU/mL, *E*_2_ 235.2, and P 14.2 pg/mL A routine blood test showed the following: white blood cells (WBC) 20.34 × 10^6^/mL; neutrophil ratio 83.7%; hemoglobin 103 g/L; and erythrocyte sedimentation rate 110 mm/h. Transvaginal sonography (TVS) revealed no clear echo indicating the presence of the pregnant sac in the uterine cavity or the annex area. After a review of the patient’s history, a TB recurrence was suspected and confirmed by the Gansu Province TB specialized hospital.

The patient received a four-drug chemotherapy, but still had a low fever. On day 28, a new TVS showed that there was no pregnancy sac in the uterine cavity, but a cyst image about 11 × 10 mm appeared at the level of the cervix; no definite embryo image or heartbeats could be detected. Also, a low-density mass measuring 79 × 70 mm became visible on the right ovary. At this point a diagnosis of cervical pregnancy was suspected, combined with a right ovarian tuberculotic cyst.

A decision was then made to terminate the pregnancy, and the gestational sac was injected with 50 mg of methotrexate through the cervical canal; in addition, 50 mg of mifepristone tablets were administered three times daily. 4 days later, the gestational sac was expelled. A pathological examination confirmed that it was indeed a cervical pregnancy. 1 week later, she underwent laparoscopy for the right salpingectomy and oophorectomy. The specimen contained about 150 mL of purulent discharge, which was sent for culture; the cystic wall was sent for histologic examination. Pathology demonstrated chronic nonspecific inflammation in the oviduct and ovarian suppurative inflammation. Cultures for acid-fast bacilli were negative.


**Informed consent:** The case report is from Reproductive Medicine Specialist Hospital of the First Hospital of Lanzhou University in China. An institutional review board approval was not required in this case. The woman and her husband signed an informed consent form after being properly informed of the situation.

## 3 Discussion

### 3.1 Pregnancy and TB

The special physiological state and changes in the endocrine environment in pregnant women are closely related to the occurrence and development of TB. Such changes include autonomic dysregulation, endocrine and metabolic disorders, increased ovarian hormones estrogen and progesterone, hyperemia in the lung, hyperthyroidism, increased metabolic rate, and increased energy consumption; also, an obvious increase in secretion of adrenocortical hormone results in an increase in capillary permeability. All these symptoms are conductive to reactivation of latent *M. tuberculosis*, leading to the recurrence and even worsening of TB. There is no significant difference between the symptoms of pregnancy complicated by TB and solitary TB, and pregnancy symptoms often overlap making it difficult to diagnose, delaying appropriate treatment [6]. Medium and high fever symptoms tend to raise the vigilance of clinicians and patients, but chest X-ray and chest CT-scan are not suitable for pregnant patients. Most clinicians traditionally first consider pulmonary infection, based on certain clinical signs and symptoms, history of disease, and laboratory studies. They then provide anti-infection treatment with common antibiotics for a period of time, which also delays early diagnosis of TB [7].

### 3.2 IVF and TB

IVF offers infertility couples new hope of having a baby. However, steps of IVF treatment differ greatly from those of a natural pregnancy. A large number of steroids hormone and gonadotropin are used during IVF, resulting in both a rapid increase of female estrogen and progesterone to a level significantly higher than the normal physiological level, which in turn suppresses t-lymphocyte proliferation. Immune response of the Th1 cell is reduced, while that of the Th2 cell is enhanced, resulting in Th1/Th2 imbalance [8]. As a consequence, TB infection becomes more likely and recurrant in women conceiving by IVF compared to those conceiving naturally.

In most cases, for those patients whose infertility is immunologic or unexplained, repeated implantation failures often require oral administration of prednisone during IVF to improve endometrial receptivity: that will increase the risk of latent and static *M. tuberculosis in vivo* resurgence or outbreak, leading to the recurrence of TB [9].

### 3.3 Early prevention and diagnosis of TB during IVF

In clinical work, our usual practice is to do hysterosalpingography and related examinations to explore the physiological reason for infertility, but only rarely are routine endometrial and oviduct mucosal histological examination to exclude TB infection done [10]. Therefore, we propose that it is necessary to perform routine screening such as chest CT, pelvic ultrasonography or CT examination during IVF to detect latent *M. tuberculosis* infection early and provide appropriate intervention, especially in locations with a high incidence of TB. Highly suspicious cases should undergo further examinations such as laparoscopic examination, acid-fast bacilli sputum smear and mycobacterial culture, pathological examination of biopsy samples, and *T*-SPOT TB examination [11]. Also, prophylactic anti-mycobacterial treatment should be administered when necessary to avoid adverse outcomes. Only her condition is stable should an infertile female enter the IVF cycle.

Moreover, it is suggested that pregnancy should be terminated as soon as active TB is confirmed. Preventing the birth of a neonate affected by congenital TB is desirable, and estrogen and progestogen levels decrease as a result of termination, which will reduce the negative impact of it on immune function and favor the patient’s recovery [12]. However, attention should be paid to administering systemic treatment with anti-TB drugs at least 2 weeks before induced abortion [13]. One reason is to prevent surgical trauma aggravated by TB conditions, and the other reason is to avoid the risk of *M. tuberculosis* spreading throughout the body. If couples decide to continue pregnancy, close follow-ups should be scheduled by both obstetrics and infectious disease specialists during anti-TB treatment to monitor fetal conditions and to minimize the risk of congenital TB [14].

### 3.4 Diagnosis and treatment of cervical pregnancy

Cervical pregnancy is a rare form of ectopic pregnancy, with an incidence of 0.001–0.04%, accounting for about 0.1% of all ectopic pregnancies [15,16]. Cervical pregnancy occurs in a special anatomical site, and the etiology is complex. Some iatrogenic interventions may increase its incidence, such as cervical dilatation, induced abortion, and IUD use., which may lead to cervical trauma, endometrial injury, and an abnormal environment of the uterine cavity [17]. Furthermore, the cervix is mainly composed of vascular-rich connective tissue and contains less muscle tissue. Uterine isthmus contractility is comparatively weak when abortion occurs. If the process is not appropriately managed, life-threatening uncontrolled bleeding can occur.

The incidence of cervical pregnancy is considerably low, and a unified standardized treatment plan is not available at present. Consequently, once cervical pregnancy is diagnosed, it is necessary to actively formulate treatment plans and terminate pregnancy as soon as possible to avoid the risk of fatal bleeding. Methotrexate, by inhibiting dihydrofolate reductase, causes embryonic tissue necrosis, drop out, and even resorption of the sac [18]. Mifepristone acts as a progesterone antagonist with anti-progestinic activity, causing villous degeneration and embryo necrosis [19]. Simultaneously, because the patient had experienced recurrent episodes of GTB and had multiple previous surgeries, the patient’s preferences with respect to future fertility and the desire to have a child should be reevaluated. In the present case study, an ultrasound-guided local injection of methotrexate with concurrent oral mifepristone treatment plan was clinically developed. In the end, the gestational sac was expelled 4 days later with minimal blood loss. Therefore, we think this treatment protocol may be a good alternative in such patients: it presents the advantage of being less invasive, more acceptable to patients, and is cost- and time-effective.

## 4 Conclusion

Screening for TB in infertility patients undergoing assisted reproduction is necessary, especially in primary infertility women from regions with high incidence of TB. For latent TB-infected patients more clinical data would be needed to decide whether to employ short-term anti-TB treatment without affecting the IVF treatment to reduce the incidence of TB during pregnancy.
